# A Bibliometric and Visualization Analysis of Mindfulness and Meditation Research from 1900 to 2021

**DOI:** 10.3390/ijerph182413150

**Published:** 2021-12-13

**Authors:** Yuzheng Wang, Lingqiu Liao, Xiaoxiao Lin, Yabin Sun, Ning Wang, Jinyan Wang, Fei Luo

**Affiliations:** 1 CAS Key Laboratory of Mental Health, Institute of Psychology, Chinese Academy of Sciences, Beijing 100101, China; wangyz@psych.ac.cn (Y.W.); liaolq@psych.ac.cn (L.L.); linxx@psych.ac.cn (X.L.); sunyb@psych.ac.cn (Y.S.); wangn@psych.ac.cn (N.W.); wangjy@psych.ac.cn (J.W.); 2 Department of Psychology, University of Chinese Academy of Sciences, Beijing 100049, China; 3 Shanghai Key Laboratory of Mental Health and Psychological Crisis Intervention, School of Psychology and Cognitive Science, East China Normal University, Shanghai 200062, China

**Keywords:** mindfulness, meditation, CiteSpace, VOSviewer, bibliometric, well-being, health

## Abstract

This study comprehensively summarizes research in the field of meditation, especially mindfulness meditation from 1900 to 2021, by analyzing the knowledge map through CiteSpace and VOSviewer software. Using “mindfulness *” or “meditation *” as the topic, articles included in the Science Citation Index Expanded and Social Sciences Citation Index were searched in the web of science core database, resulting in the selection of 19,752 articles. Over half a century ago, Deikman published the field’s first article in the Journal of Nervous and Mental Disease in 1963, and publications have soared in subsequent decades. The USA is in the core position in terms of global collaboration, total publication numbers, and total citations. The *Mindfulness* journal ranked first for the most published articles and citations. “The benefits of being present: Mindfulness and its role in psychological well-being,” written by Brown and Ryan, was the most cited article. Mindfulness, meditation, depression, intervention, stress reduction, stress, and anxiety are the top co-occurrence keywords. The timeline of cluster analysis discloses that before 2010, hypertension, cancer, mindfulness, generalized anxiety disorder, and other topics received great attention. In the decade since 2010, scholars have shown interest in meta-analysis, attention, and self-assessment, and keen attention to mindfulness-based interventions. These findings provide an important foundation to direct future research.

## 1. Introduction

Meditation is comprised of a family of self-regulation practices that train attention and awareness to bring mental processes under greater voluntary control to thereby foster general mental well-being and development, and/or specific capacities such as calmness, clarity, and concentration [1]. Among the various categories of meditation, mindfulness, as “the awareness that emerges through paying attention on purpose, in the present moment, and nonjudgmentally to the unfolding of experience moment by moment,” has generated increasing scholarly attention [2]. The number of mindfulness and meditation studies has expanded in recent years. Multiple reviews and meta-analyses have analyzed and summarized the effects of meditation on behavior and brain imaging in clinical and non-clinical subjects, and have demonstrated significant physical and mental health benefits [3,4,5,6]. In addition, mindfulness is a popular practice. According to a survey conducted by the U.S. National Institutes of Health, 8% of American adults practiced meditation in 2012 [7].

In recent decades, the research on mindfulness and meditation has developed rapidly, and a variety of topics have emerged [3,6]. For example, research hotspots range from stress reduction [8,9,10] and prevention of depression recurrence [11,12], to attention improvement [13,14,15], pain analgesia [16], and even academic achievement improvement [17]. Despite the rapid development of mindfulness and meditation, it should be noted that there are still some conceptual and methodological issues in research on mindfulness and meditation, which have an important bearing on the future scientific understanding of mindfulness and meditation [18].

Therefore, an analysis of the development, current status, and the relationships between key points and important events of the mindfulness and meditation literature is essential to inform the direction of future research.

Bibliometrics is the cross-disciplinary science of quantitative analysis of all knowledge carriers by mathematical and statistical methods [19]. It is not only a relatively mature and important branch of intelligence science [20,21], but also a discipline that is based on quantitative analysis through the intersection and combination of philology, information science, mathematics, and statistics in a specific area [22]. With the rapid development of computer, network, and information technologies, the information of knowledge data becomes more open. Consequently, the bibliometrics method is used to describe, evaluate, and predict the status and development trend of scientific and technological research in certain fields [23]. The most obvious advantage of bibliometrics is that it allows scholars to study a specific research area by analyzing citations, co-citations, geographical distributions, and word frequencies to draw useful conclusions. Heretofore, bibliometrics has been widely used in hotspot research [24], co-authorship analysis [25], co-citation analysis [26], and the development of the whole subject fields [27].

The aim of this study was to reveal the inner structure pattern and citation landscape of mindfulness and meditation publications from a general perspective. CiteSpace and VOSviewer were used to analyze annual publication outputs, publication distributions by country and institution, author productivity, and collaboration patterns to provide an informed perspective on the evolution and development of mindfulness and meditation as a scientific discipline. Moreover, analyses of document co-citation networks, research clusters, and reference citation bursts were performed to offer insights into research topics and trend evaluations over time from different perspectives, thereby facilitating future research.

## 2. Methods

### 2.1. Search and Inclusion Criteria

Science Citation Index Expanded (SCI-E) and Social Sciences Citation Index (SSCI) articles from 1900 to 2021 (4 March 2021) were searched, with “mindfulness *” or “meditation *” as the theme, in the Web of Science Core Collection. Considering that mindfulness is an important part of meditation classification and the focus of the current research, and there is a mixture of mindfulness and meditation concepts in some studies [18,28,29], “mindfulness *” was especially emphasized when screening keywords. Only the documents of article or review type were included, while other document types such as meeting abstracts, letters, book reviews, and corrections were excluded. This search yielded 19,752 articles. Among the selected articles (19,752), a total of 561 papers were non-English. In the process of bibliometric analysis, the title, abstract, and keywords are the main analysis terms, and these sections are described in English, so they will not affect the final results.

### 2.2. Data Analysis

CiteSpace 5.3.R4 (Philadelphia, PA, USA) and VOSviewer 1.6.16 (Rotterdam, The Netherlands) were used for knowledge mapping analysis. CiteSpace is a Java application for analyzing and visualizing co-citation networks [30]. It supports structural and temporal analyses of a variety of networks, including collaboration networks, author co-citation networks, and document co-citation networks. It also supports networks of hybrid node types such as terms, institutions, and countries, and hybrid link types such as co-citation, co-occurrence, and directed citing links [30]. In this study, CiteSpace was used to analyze and visualize the reference co-citation clusters, timeline view of clusters, and the citation bursts.

VOSviewer is a software tool developed for constructing and viewing bibliometric maps. It constructs a map based on a co-occurrence matrix [31].

In the visualizations, one node represents a project (such as an author or a country), the size of the node reflects its importance (e.g., number of papers published by the project), and the color of the node signifies the group according to the default clustering method. Network connections denote collaborations between projects. The line width represents the intensity of collaboration. In this study, we used VOSviewer to make country collaboration, inter-institution collaboration, co-authorship, and co-citation networks.

Before analyzing the results, all the inconsistencies in the bibliographic data were standardized and corrected. These inconsistencies appeared due to errors in the process of capturing the WOS data, abbreviations, and mistakes made by the authors in the references. For example, to prevent classification errors, we uniformly revised the expressions cognitive behavior therapy, cognitive behaviour therapy, cognitive behavioural therapy, cognitive-behavior therapy, and cognitive behavioral therapy to cognitive-behavioral therapy. The inconsistencies were checked by the first author. See Appendix A Table A1 for details.

## 3. Results

### 3.1. General Analysis of Publications

#### 3.1.1. Annual Comparative Analysis of Mindfulness and Meditation Publications

Meditation emphasizes mental development, such as bhavana (mental cultivation) in Buddhism and lien-hsin (refining the mind) in Taoism. In Western definitions, meditation is a self-regulation strategy with a particular focus on training attention [1]. The first meditation article was published by Deikman in the *Journal of Nervous and Mental Disease* in 1963 [32]. Eight subjects participated in the experiment, where four performed meditation for twelve sessions and the other four performed brief meditation control procedures. The participants learn about contemplative meditation, and concentrate on a blue vase without analyzing the different parts of the vase by thinking a series of thoughts about the vase. Results showed phenomena common to all subjects, such as perception of the vase (e.g., more vivid), time-shortening (e.g., feel less time elapsed), conflicting perceptions (e.g., the vase filled my visual field/It did not fill the field by any means), development of stimulus barriers (e.g., increase in ability to keep out distracting stimuli), personal attachment to the vase, and pleasurable quality (e.g., pleasurable, valuable, and rewarding). For the first time, this article described the meditation training methods and the subjects’ experience in great detail.

In addition to Deikman’s research, Kabat-Zinn played an important role in the promotion of meditation. Among the top 10 most frequently cited articles, Kabat-Zinn contributed 3 articles (cited 1804, 939, and 374 times). See Appendix B and Appendix C (Table A2 and Table A3) for details. Kabat-Zinn’s pioneering contribution is that he has established a systematic eight-week mindfulness-based stress reduction (MBSR).

**Figure 1:**
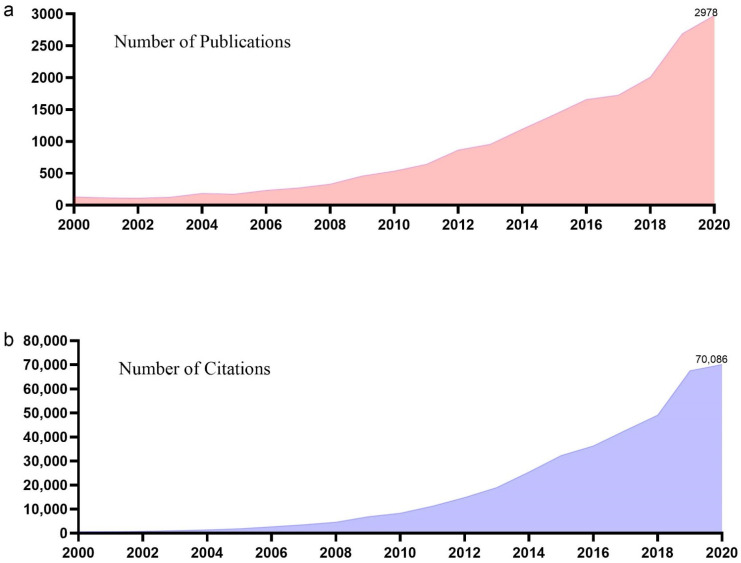
Mindfulness meditation publications (**a**) and citations (**b**)—annual distributions.

During the century from 1900 to 1999, only 625 meditation articles were published (5 in the 1960s, 174 in the 1970s, 115 in the 1980s, and 331 in the 1990s). Figure 1 depicts the numbers of publications and citations of mindfulness and meditation articles since 2000. The number of global mindfulness and meditation articles included in SCI-E/SSCI increased annually, from 131 per year in 2000 (0.70% of the total publications) to 2978 per year in 2020 (15.83% of the total publications). The number of articles published annually increased by over 30% during five particular years (46.46% in 2004, 33.91% in 2006, 37% in 2009, 35.31% in 2012, and 34% in 2019). The number of citations included in SCI-E/SSCI from 2000 to 2020 also increased annually, from only 517 (0.13% of total citations) in 2000 to 70,086 (17.17% of total citations) in 2020. Annual increases of at least 30% were observed in more than half of the included years.

**Figure 2:**
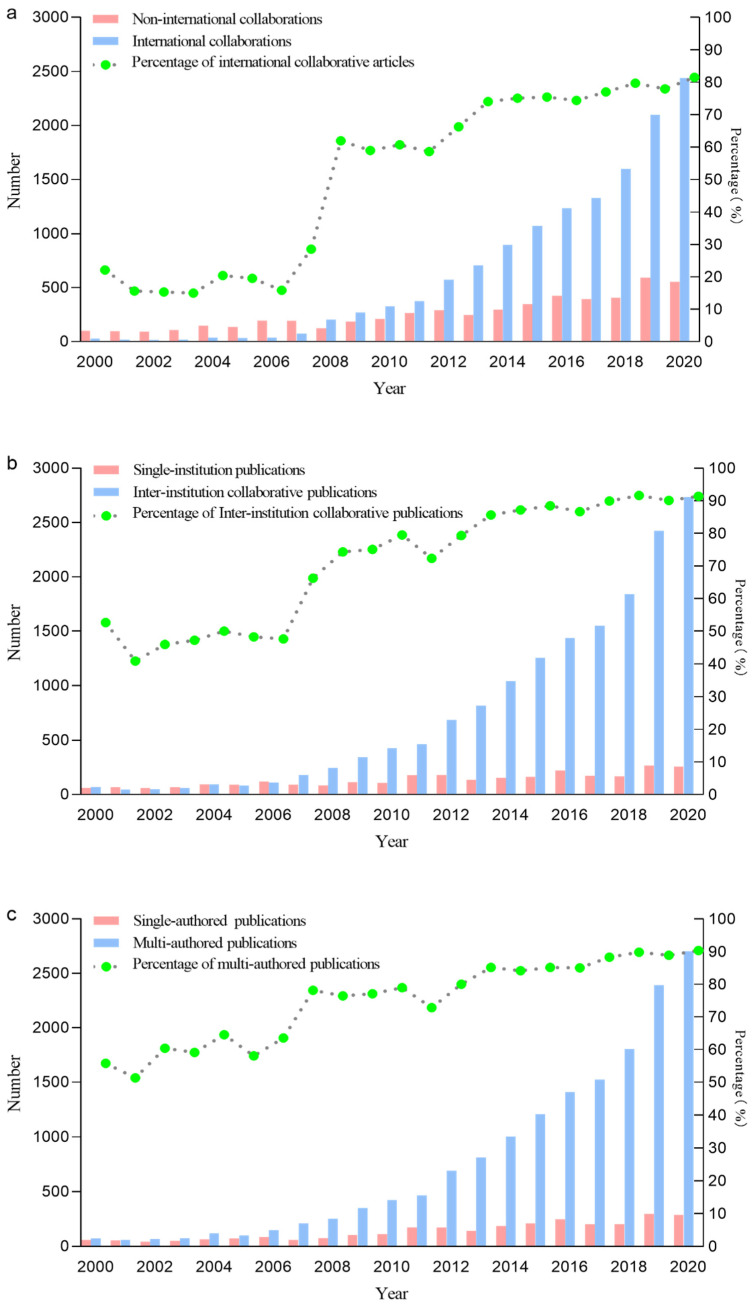
Collaborations of countries (**a**), institutions (**b**), and authors (**c**) in published articles.

Figure 2a exhibits the changing trends of international and non-international collaborative publications, as well as percentages of international collaborative articles. Collaborations of institutions and authors are presented in Figure 2b,c, respectively. Increasing rates of collaborative research among countries (81.48%), institutions (91.38%), and authors (90.34%) reflect the prevailing trend. The proportion of collaborative articles accelerated most rapidly during 2007–2008, and continues to rise.

#### 3.1.2. Most Cited Articles

Table 1 shows the 15 most cited mindfulness and meditation articles. “The benefits of being present: Mindfulness and its role in psychological well-being,” published in the *Journal of Personality and Social Psychology* (JPSP) by Brown and Ryan (2003) [33], was cited most frequently (4585 citations), followed by “Assessment and Clinical Psychology-Science and Practice” by Baer et al. (2006) [34] and “Mindfulness: A Proposed Operational Definition” by Bishop et al. (2004) [35], ranking second and third with 2849 and 2597 citations, respectively. The two most frequently cited articles described the development and psychometric properties of the Mindful Attention Awareness Scale (MAAS) and the Five-Facet Mindfulness Questionnaire (FFMQ), respectively. The third most cited article proposed a two-component model and an operational definition of mindfulness.

Among the top fifteen most cited publications, three authors contributed two articles each. For example, Brown published two highly cited articles in JPSP (4585 citations) as well as in *Psychological Inquiry* (1370 citations). Baer published highly cited articles in *Assessment* (2849 citations) and in *Clinical Psychology-Science and Practice* (CPSP, 2004 citations), while Kabat-Zinn wrote impactful papers in CPSP (2553 citations) and *General Hospital Psychiatry* (1666 citations).

The most cited source journal is JPSP, which publishes original papers in all areas of personality and social psychology, including attitude and social cognition, interpersonal relations and group processes, as well as personality processes and individual differences. Three highly cited articles were from CPSP (597, 2553, and 2004 citations), the official publication of the American Psychological Association Division 12, the Society of Clinical Psychology. The journal publishes papers regarding developments in the science and practice of clinical psychology and related mental health fields. Two articles were from the *Journal of Consulting and Clinical Psychology* (JCCP, 1682 and 1660 citations), which publishes articles regarding prevention and treatment in all areas of clinical and clinical–health psychology, and especially on topics that appeal to a broad readership. The journal addresses a wide range of topics that include epidemiology, the use of psychological services, as well as healthcare economics of behavioral therapy.

#### 3.1.3. Most Productive Countries/Territories

The total number of publications, total citations, and CP (citations/publication) can be used to analyze the impact of papers published in leading countries. The countries producing the most publications on mindfulness and meditation were the USA, England, Australia, Canada, Germany, the People’s Republic of China, The Netherlands, Spain, Italy, and India (Table 2). The USA has produced 47.99% of papers, higher than the percentage of papers published by the other top nine countries (45.51%). The citation frequency, particularly the CP, is an important indicator of research quality. The USA (265,856) generated far more total citations than England (50,257), followed by Canada (43,312). Canada ranked first in CP (31.25), followed by the USA (28.05), England (27.40), the Netherlands (26.10), and Germany (25.16).

#### 3.1.4. Most Productive Institutions

Nine of the ten most prolific institutions in the mindfulness and meditation field were located in the USA. The top three institutions were Harvard University, the University of Toronto, and the University of Washington. The top three institutions for total citations were Harvard University (20,074), the University of Massachusetts (17,572), and the University of Toronto (14,657), while articles from the University of Massachusetts (86.56), the University of Toronto (56.81), and the University of Wisconsin (51.24) generated the highest CP (Table 3).

#### 3.1.5. Most Productive Journals

Table 4 shows the total number of articles published in the top 20 journals that address mindfulness and meditation, as well as the number of citations generated by the particular journal’s articles, and the consequent CP. Although the *Mindfulness* Journal began recently, in 2010, as a journal dedicated to the mindfulness field, it has published the largest number of articles (1288), followed by *Frontiers in Psychology* (442) and *Annals of Behavioral Medicine* (269). *Mindfulness* ranked first in total citations (15,208), followed by *Behavior Research and Therapy* (9601 citations) and *Personal and Individual Differences* (6084 citations). *Behavior Research and Therapy* ranked first in CP (76.81), followed by the *Journal of Clinical Psychology* (54.39) and *Consciousness and Cognition* (38.33). Three articles were cited more than 1000 times, and these were Hayes et al. (2006) [36] from *Behavior Research and Therapy* (2327 citations), Shapiro et al. (2006) [42], *Journal of Clinical Psychology* (1273 citations), and Davidson et al. (2003) [43] from *Psychological Medicine* (1237 citations).

### 3.2. Analysis of Collaborations

**Figure 3:**
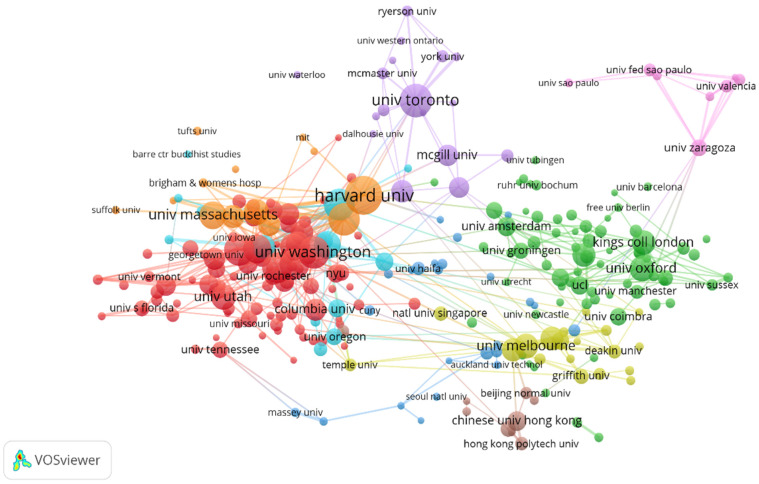
Inter-institution collaboration in mindfulness/meditation.

**Figure 4:**
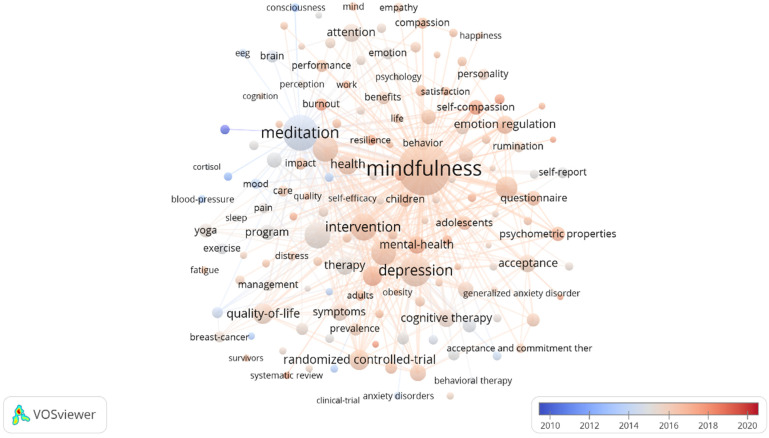
Keyword co-occurrence analysis network diagram in mindfulness/meditation.

The results of our analysis of collaborations among countries (regions), institutions, and authors are presented in Figure 3 and Figure 4. In the visualizations, one node represents a project (such as an author), the size of the node reflects the number of papers published by the project, and the color of the node signifies the group according to the default clustering method. Network connections denote collaborations between projects. The line width represents the intensity of collaboration.

#### 3.2.1. Analysis of Collaborative Networks among Countries/Territories

**Figure 5:**
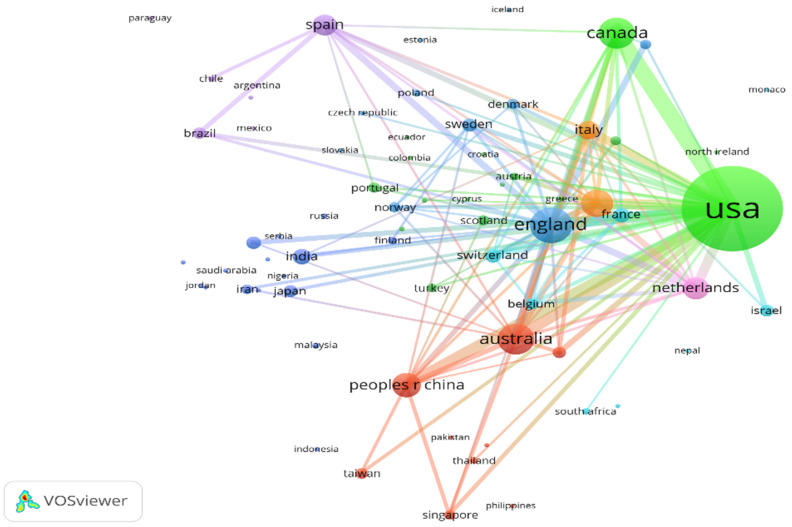
Collaboration among countries (regions) in the field of mindfulness meditation.

Link strength, literature quantity, and citations are the indicators of collaborative degree. Figure 5 displays the collaborative network between countries (regions) in the mindfulness and meditation field (the threshold of link strength is set to 10). From the perspective of link strength, the USA (24,309,335 articles, 26,2811 citations) has been the center of global collaboration in this field, and its international collaborative activity is much higher than that of other countries. The USA was followed by England (12,791,805 articles, 49,712 citations), Germany (8,031,114 articles, 28,146 citations), Australia (7,991,408 articles, 24,118 citations), Canada (7,251,368 articles, 43,154 citations), the Netherlands (584,731 articles, 18,971 citations), China (582,908 articles, 10,127 citations), Spain (472,653 articles, 7079 citations), Italy (462,653 articles, 9371 citations), Switzerland (358,305 articles, 8744 citations), and France (271,328 articles, 3998 citations).

#### 3.2.2. Analysis of Collaborative Networks among Institutions

The number of literature access was set to 30, and 264 of the 10,495 institutions meeting the standards were displayed. Figure 4 shows the inter-institution collaborative network (the threshold of link strength shown in the setting is 5). In terms of link strength, Harvard Medical School (strength 442,245, 2269 citations) was at the center of global collaboration, followed by Massachusetts General Hospital (strength 352,180, 5367 citations), Harvard University (strength 344,318, 17,747 citations), University of California, San Francisco (strength 334,237, 6644 citations), Brown University (309,209, 5064 citations), Stanford University (297,196, 7542 citations), the University of North Carolina System (288,227, 9179 citations), the University of Toronto (286,257, 14,639 citations), the University of Melbourne (269,170, 4200 citations), and the University of Massachusetts (249,201 articles, 17,207 citations).

### 3.3. Keyword Co-Occurrence Network Analysis

Keywords represent the highly concentrated content of literature research. The co-occurrence frequency of keywords can reflect dynamic trends of mindfulness and meditation research. Keywords with high frequencies reflect the focus of the research field. They identify advanced research methods, hot issues to be solved urgently, or academic topics of interest in particular timeframes.

Figure 5 shows the keyword co-occurrence analysis network diagram. All keywords were included in the analysis. Among all 30,430 keywords, the top 150 were displayed for mapping. The top 20 co-occurring keywords are shown in Table 5. Mindfulness and meditation were the most frequent, followed by depression, intervention, stress reduction, stress, and anxiety. The highly frequent use of keywords such as quality of life, health, emotion regulation, therapy, cognitive therapy, mental health, symptoms, and cognitive behavioral therapy reflect wide therapeutic applications of methods based on mindfulness, such as mindfulness-based cognitive therapy (MBCT) and mindfulness-based stress reduction (MBSR), that are used to prevent recurrent depression and to reduce stress. In addition, validation appeared frequently in keywords, such as the validity test of measurements of mindfulness. Keywords also reflected a large number of randomized controlled trial studies and meta-analyses to report empirical demonstrations of the clinical utility of mindfulness and meditation. In addition, attention and acceptance are also research foci. On one hand, mindfulness and meditation can improve the quality of attention and acceptance; on the other hand, attention and acceptance are the two core elements of mindfulness [60,61]. 

#### 3.3.1. Co-Citation Analysis

If two items (such as articles) are cited together in the references of a cited item, they are co-cited. The number of co-citations can be used to measure the similarity and correlation between articles. Journal co-citations, article co-citations, and keyword co-citations can be analyzed according to different project contents.

#### 3.3.2. Journal Co-Citation Analysis

Through the analysis of co-cited documents, we can obtain a knowledge base of a particular field. The research frontier is composed of a collection of cited literature that comprises these knowledge bases.

**Figure 6:**
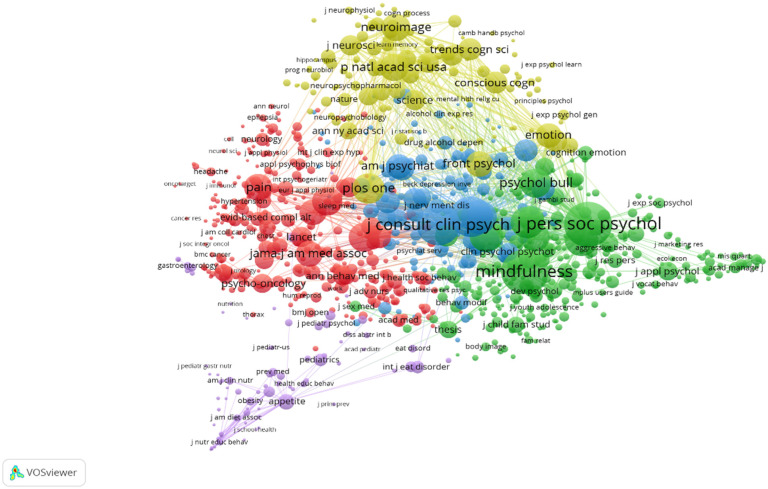
Co-citation analysis network of journals in the field of mindfulness meditation.

The VOSviewer identified 96,779 journals. Table 6 displays the results of our journal co-citation analysis. Figure 6 shows the co-citation connectivity between journals. The Journal of Personality and Social Psychology was the most frequently cited journal, and is in the core position of the network. The Journal of Consulting and Clinical Psychology ranked second.

#### 3.3.3. Reference Co-Citation Analysis

**Figure 7:**
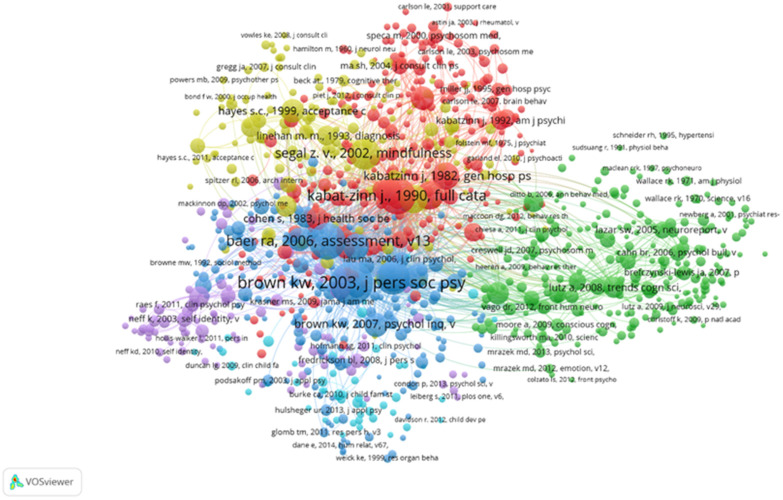
Co-citation analysis network diagram of references in the field of mindfulness meditation.

A co-citation relationship is defined by the simultaneous citations of two papers in a third paper [62]. Reference co-citation analysis is an important means to detect the structure and evolutionary path of a specific domain. Figure 7 shows the top 1000 of 412,783 tabulated references. Among the top 20 most frequently cited articles, Kabat-Zinn and Baer contributed 4 articles each, while Brown authored 2 articles. Seven publications were review articles or meta-analyses [2,13,37,38,39,41]. Another six articles were classic papers written by leaders in the field introducing therapeutic interventions such as the MBSR [8,9,10], MBCT [11,12], and acceptance and commitment therapy (ACT) [63]. The other five articles reported the development of mindfulness questionnaires, such as the MAAS [36], FFMQ [34,64], and the Kentucky Inventory of Mindfulness Skills [65]. Stress and stress reduction are important co-occurrence keywords (Table 7), so the development of the Perceived Stress Scale (PSS) is also an important co-citation article [66]. There are also two articles on the mechanism of mindfulness [42,67].

**Figure 8:**
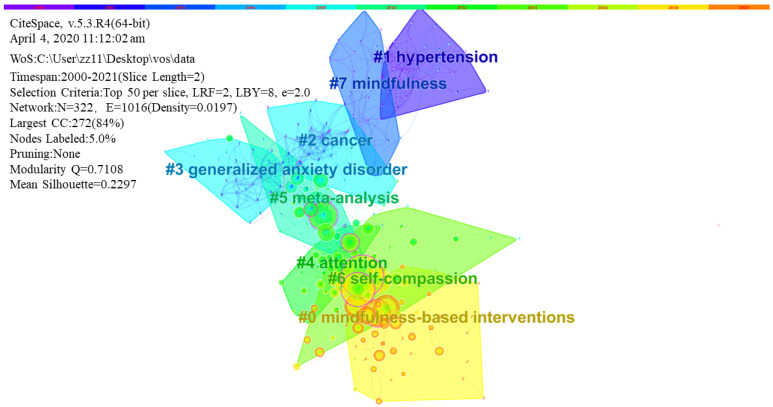
Reference co-citation clusters in mindfulness meditation.

**Figure 9:**
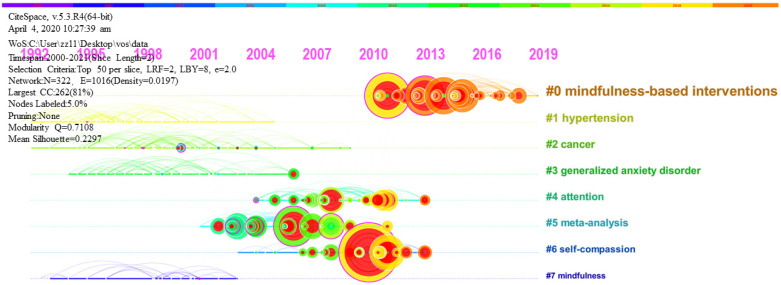
Timeline view of the temporal progression of activity in each cluster in the mindfulness and meditation field.

Next, the literature was divided into clusters. The log-likelihood test algorithm was used to extract nominal terms from the keywords of the cited literature and to name the clusters (Figure 8). To better reflect the time characteristics impacted by clustering, Figure 9 places the documents of the same cluster on the same horizontal timeline. The number of documents in each cluster can be clearly represented in the timeline view. The more articles in a cluster, the more important the clustering field. The timespan of documents in each category can also be obtained to further reflect the time characteristics influenced by clustering.

### 3.4. Burst Detection Analysis

Publications receiving a surging citation frequency reflect emerging trends in scientific research fields. Citation bursts suggest increased scholarly attention to the corresponding publications. Table 8 lists the top 30 references with the strongest citation bursts cited by INS publications. Green color indicates the years since the article was published. Red color indicates the years with citation bursts.

CiteSpace provides burst detection that can detect large changes in the number of citations in a particular timeframe, and is used to identify the declining or increasing use of a topic word or keyword. Table 8 presents the results of the emergent analysis of the top 30 publications. The red segments show the starting and ending times of particular literature bursts. The strongest burst citation was exhibited by an article by Brown et al. [33] that reported the development of the MAAS questionnaire to explore the benefits of mindfulness in improving psychological well-being. The next strongest bursts were associated with two articles by Baer that introduced the FFMQ questionnaire and discussed the five elements of mindfulness (2006) [34], and that reviewed the clinical utility of mindfulness training (2003). The earliest article in our burst detection analysis was a review by Teasdale et al. [12], one of the developers of MBCT, who utilizes mindfulness cognitive therapy to prevent recurrent depression.

## 4. Discussion

Our study provided a comprehensive perspective of the evolution and development of mindfulness and meditation literature. By using bibliometric methods and customized text mining techniques, we analyzed distribution (including distributions of countries/territories, institutions, and journals), collaboration (including international, interinstitutional, and inter-author collaborations), and document co-citations (including keyword co-occurrence networks, journal co-citations, reference co-citations, and reference citation bursts).

First, the number of publications and citations may reflect the overall development of the mindfulness and meditation field. No papers on mindfulness and meditation were published until over half a century ago, when Deikman [32] published the field’s seminal article in the *Journal of Nervous and Mental Disease*. Consequently, mindfulness and meditation research have a long history. However, the numbers of mindfulness and meditation-related publications fluctuated at low levels during the 20th century. Since 2004, the number of publications has increased by more than 30% every few years. The citation rate has increased by more than 30% in over half of the years since 2002. At the same time, the prevailing trend in mindfulness and meditation literature has been towards increased collaboration among countries, institutions, and authors.

Second, from the perspective of distribution, the USA has been the center of global collaboration, and its strength of cooperation has been much higher than that of other countries. The USA also has led other countries in total numbers of publications and citations. However, Canada ranked first in the CP. From the perspective of research institutions, Harvard University ranked first in total numbers of publications and citations, while the University of Massachusetts ranked first in the CP. Nine of the top ten institutions in the global field of mindfulness and meditation are located in the United States. Harvard Medical School has been at the center of inter-institution collaboration. Taken together, our data indicate that the USA is in the core position in the field of mindfulness and meditation.

Third, in terms of the influence of journals and authors, *Mindfulness* ranked first for the most published articles and citations. However, *Behavior Research and Therapy* ranked first in the CP. From the perspective of journal co-citation analysis, JPSP has the most co-citations and the largest link strength, followed by *Mindfulness*. Brown and Ryan are the most cited authors, due to multiple citations of their article: “The benefits of being present: Mindfulness and its role in psychological well-being” [33].

Fourth, keyword analysis disclosed that in general, physical and mental health disorders such as depression, anxiety, stress, emotion regulation, and quality of life have been the primary research foci in the field of mindfulness and meditation, which reflects its wide therapeutic applications. At the same time, a large number of randomized controlled trials and meta-analyses have been completed. Researchers have also paid attention to validity of measurement tools and the mechanism of mindfulness and meditation.

Fifth, JPSP, *Mindfulness*, and JCCP are the leading journals in the field of mindfulness and meditation. The common concerns of scholars include reviews and meta-analyses of the clinical utility of mindfulness and meditation, and these include the classic articles by the developers of MBSR, MBCT, and ACT, and mindfulness-related questionnaires. The timeline of cluster analysis discloses that before 2010, hypertension, cancer, mindfulness, generalized anxiety disorder, and other topics received great attention. In the decade since 2010, scholars have shown interest in meta-analysis, attention, and self-assessment, and keen attention to mindfulness-based interventions. The development of MAAS and FFMQ questionnaires and the studies of the clinical efficacy of mindfulness and meditation showed the strongest bursts. A review of the effect of MBCT on preventing recurrent depression showed the earliest citation burst.

Recently, another article using the bibliometric method to analyze mindfulness hotspots was published [81]. In contrast, the inclusion criteria of the two articles are different, and the final results are somewhat different. For example, Baminiwatta and Solangaarachchi (2021) [81] searched for articles from 1966 to 2021 in the WOS core collection, with mindfulness as the topic. Finally, 16,581 articles were included. In this study, SCI-E and SSCI articles from 1900 to 2021 (4 March 2021) were searched, with “mindfulness *” or “meditation *” as the topic in the WOS core collection, and 19,752 articles were included. Therefore, Baminiwatta and Solangaarachchi (2021) [81] found that the first mindfulness article was “Mindfulness of Perception” published in 1966, and we found that the first article in the meditation field was “Experimental meditation” in 1963. Meditation and mindfulness are closely related, so the two articles can complement each other from different views.

## 5. Implications and Future Directions

Citation bursts reflect the emerging trends in scientific research fields and suggest the future directions. Tang et al., (2015) [68] showed the strongest citation bursts among the recent emerging ones. The authors described the neuroscience of mindfulness meditation and recommended that further research with longitudinal, randomized, and actively controlled research designs and larger sample sizes are needed to advance the understanding of the mechanisms of mindfulness and meditation [68,73].

It is essential to pay attention to misinformation and poor methodology in previous mindfulness studies, with a particular focus on assessment, mindfulness training, possible alternative effects, and intersection with brain imaging in the future [28]. Scholars believe that future research in meditation would benefit from addressing the remaining methodological and conceptual issues [70,73,75].

Furthermore, effectiveness studies that carefully consider how to reach out to communities of need with evidence-based, cost-effective, and sustainable mindfulness interventions are needed in the coming years [79]. The timeline of analysis of clusters also confirmed that mindfulness-based interventions are still an ongoing trend.

## 6. Limitations

Although meditation can be roughly divided into focused attention meditation and open monitoring meditation, it is difficult to separate specific meditation techniques (e.g., Chan, Taichi, Baduanjin qigong), so the subcategories of meditation were not compared in this study.

The names of the clusters were obtained by extracting terms from the keywords of the references through the log likelihood test algorithm. Since over 19,752 articles were identified, it was impossible for the authors to refine and analyze the content of each article. Since the articles were not randomly checked as to whether they are related to the topic, this may be problematic because some terms, such as meditation, have multiple meanings in English, some of which are irrelevant. Another major limitation is that the search was limited to databases (SCI-E, SSCI) that mainly include English publications, excluding most journals published in other languages. This will bias the results. For example, if Chinese or Korean databases were included, the results may be very different from those of a large number of papers in East Asia.

As an interdisciplinary science of quantitative analysis, bibliometrics can predict the status and development trend of mindfulness and meditation. Despite these advantages, it cannot recommend which method is better through indicators such as effect size, as in meta-analysis. Furthermore, bibliometric analysis is mainly based on the abstract, title, keywords, and references, without checking the full text of the paper, which also affects the final results.

In conclusion, this study provided a comprehensive review and intuitive analysis of publications related to mindfulness and meditation from 1900 to 2021. Using two effective bibliometric tools, VOSviewer and CiteSpace, this paper analyzed status and development trends of this field from many perspectives, and may inform future research in the field of mindfulness and meditation.

## Figures and Tables

**Table 1:** The 15 most cited mindfulness and meditation articles (1900 to 2021).

Rank	Authors	Citations	Journal	Title
1	Brown & Ryan (2003) [33]	4585	Journal of Personality and Social Psychology	The benefits of being present: Mindfulness and its role in psychological well-being
2	Baer et al., (2006) [34]	2849	Assessment	Using self-report assessment methods to explore facets of mindfulness
3	Bishop et al., (2004) [35]	2597	Clinical Psychology-Science and Practice	Mindfulness: A proposed operational definition
4	Kabat-zinn (2003) [2]	2553	Clinical Psychology-Science and Practice	Mindfulness-based interventions in context: Past, present, and future
5	Hayes et al., (2006) [36]	2349	Behaviour Research and Therapy	Acceptance and commitment therapy: Model, processes and outcomes
6	Baer (2003) [37]	2004	Clinical Psychology-Science and Practice	Mindfulness training as a clinical intervention: A conceptual and empirical review
7	Grossman et al., (2004) [38]	1983	Journal of Psychosomatic Research	Mindfulness-based stress reduction and health benefits: A meta-analysis
8	Hofmann et al., (2010) [39]	1682	Journal of Consulting and Clinical Psychology	The effect of mindfulness-based therapy on anxiety and depression: A meta-analytic review
9	Kabat-zinn (1982) [8]	1666	General Hospital Psychiatry	An outpatient program in behavioral medicine for chronic pain patients based on the practice of mindfulness meditation: Theoretical consideration
10	Teasdale et al., (2000) [12]	1600	Journal of Consulting and Clinical Psychology	Prevention of relapse/recurrence in major depression by mindfulness-based cognitive therapy
11	Deci & Ryan (2008) [40]	1572	Canadian Psychology—Psychologie Canadienne	Self-Determination Theory: A macrotheory of human motivation, development, and health
12	Brown, Ryan, & Creswell (2007) [41]	1370	Psychological Inquiry	Mindfulness: Theoretical foundations and evidence for its salutary effects
13	Shapiro et al., (2006) [42]	1281	Journal of Clinical Psychology	Mechanisms of mindfulness
14	Davidson et al., (2003) [43]	1240	Psychosomatic Medicine	Alterations in brain and immune function produced by mindfulness meditation
15	Diamond & Lee (2011) [44]	1187	Science	Interventions shown to aid executive function development in children 4 to 12 years old

**Table 2:** The 10 most prolific countries/regions.

Rank	Country	Publications	Citations	Citations/Publication	Leading Institute of Each Country (Publications)
1	USA	9478	265,856	28.05	University of California System (910)
2	England	1834	50,257	27.40	University of London (393)
3	Australia	1426	24,946	17.49	University of Melbourne (170)
4	Canada	1386	43,312	31.25	University of Toronto (310)
5	Germany	1128	28,383	25.16	University of Freiburg (104)
6	People’s Republic of China	912	10,230	11.22	Chinese University of Hongkong (125)
7	The Netherlands	735	19,184	26.10	Radboud University Nijmegen (170)
8	Spain	657	7076	10.77	University of Zaragoza (100)
9	Italy	527	9389	17.82	Sapienza University Rome (86)
10	India	384	4819	12.55	All India Institute of Medical Sciences, New Delhi (44)

**Table 3:** Top 10 most productive institutions.

Rank	Institution	Publications	Citations	Citation/Publication
1	Harvard University	568	20,074	35.34
2	University of Toronto	258	14,657	56.81
3	University of Washington	254	8589	33.81
4	University of California, San Francisco	240	6924	28.85
5	University of California, Los Angeles	238	9854	41.40
6	University of Wisconsin	234	11,989	51.24
7	University of North Carolina at Chapel Hill	230	9194	39.97
8	Brown University	212	5181	24.44
9	University of Massachusetts	203	17,572	86.56
10	Duke University	184	8439	45.86

**Table 4:** Top 20 journals that published mindfulness and meditation articles (1900 to 2021).

Rank	Journal	Article Number	Citations	Citations/Article	Most Cited Article	Citations of Most Cited Article
1	Mindfulness	1288	15,208	11.81	Eberth & Sedlmeier (2012) [3]	300
2	Frontiers in Psychology	442	5039	11.40	Zenner et al. (2014) [45]	301
3	Annals of Behavioral Medicine	269	2120	7.88	Jain et al. (2007) [46]	596
4	Journal of Alternative and Complementary Medicine	246	5742	23.34	Chiesa & Serretti (2009) [47]	691
5	Personality and Individual Differences	211	6.084	28.83	Walach et al. (2006) [48]	535
6	PLoS ONE	206	5681	27.58	Lutz et al. (2008) [13]	416
7	Psychosomatic Medicine	182	5434	29.86	Davidson et al. (2003) [43]	1237
8	Psycho Oncology	174	2022	11.62	Lengacher et al. (2009) [49]	269
9	Library Journal	160	2	0.01	Lettus (2010) [50]	1
10	Journal of Clinical Psychology	144	7832	54.39	Shapiro et al. (2006) [42]	1273
11	Behaviour Research and Therapy	125	9601	76.81	Hayes et al. (2006) [36]	2327
12	Psychophysiology	123	666	5.41	Khalsa et al. (2008) [51]	134
13	Explore: The Journal of Science and Healing	114	1967	17.25	Garland et al. (2009) [52]	189
14	Complementary Therapies in Medicine	112	1521	13.58	Dalen et al. (2010) [53]	131
15	European Psychiatry	111	164	1.48	Weber et al. (2010) [54]	52
16	Frontiers in Human Neuroscience	110	3474	31.58	Vago & Silbersweig (2012) [55]	418
17	Consciousness and Cognition	107	4101	38.33	Zeidan et al. (2010) [56]	469
18	International Journal of Environmental Research and Public Health	107	411	3.84	Cocker & Joss (2016) [57]	76
19	Journal of Child and Family Studies	104	2567	24.68	Burke (2010) [58]	335
20	Journal of Contextual Behavioral Science	100	550	5.5	Francis et al. (2016) [59]	62

**Table 5:** Top 20 co-occurrence keywords.

Rank	Keywords	Co-Occurrence Frequency	Total Link Strength
1	Mindfulness	8149	43,960
2	Meditation	3952	21,263
3	Depression	3007	19,986
4	Intervention	2285	14,785
5	Stress reduction	2266	14,891
6	Stress	2123	12,731
7	Anxiety	2052	13,818
8	Validation	1607	10,080
9	Quality-of-life	1444	9131
10	Health	1422	8552
11	Randomized controlled trial	1382	8536
12	Meta-analysis	1296	8538
13	Emotion regulation	1101	7065
14	Therapy	1099	6671
15	Attention	1084	5726
16	Acceptance	1065	7273
17	Cognitive therapy	1063	7333
18	Mental health	1056	6757
19	Symptoms	987	6823
20	Cognitive-behavioral therapy	953	5669

**Table 6:** Analysis of journal co-citations.

Rand	Journal	Citations	Total Link Strength
1	Journal of Personality and Social Psychology	14,213	734,983
2	Mindfulness	13,867	690,677
3	Journal of Consulting and Clinical Psychology	11,888	629,722
4	Behaviour Research and Therapy	11,567	626,786
5	Clinical Psychology Review	8247	470,654
6	Personality and Individual Differences	7148	371,525
7	Clinical Psychology-Science and Practice	7035	344,480
8	Journal of Clinical Psychology	6914	375,828
9	Plos One	6459	405,257
10	Psychological Bulletin	6123	360,031
11	Journal of Psychosomatic Research	5973	302,685
12	Psychosomatic Medicine	5756	319,548
13	Assessment	5597	267,824
14	Neuroimage	5340	373,964
15	Proceedings of the National Academy of Science of the United States	5212	349,800
16	Pain	5147	285,924
17	Journal of Alternative and Complementary Medicine	4691	246,591
18	Behaviour Therapy	4677	256,028
19	Frontiers in Psychology	4649	275,325
20	Journal of the American Medical Association	4648	231,798

**Table 7:** Analysis of reference co-citations.

Rank	Authors (Year)	Title	Journal	Citations	Total Link Strength
1	Brown (2003) [33]	The benefits of being present: mindfulness and its role in psychological well-being	Journal of Personality and Social Psychology	3201	51,646
2 *	Kabat-Zinn (1990) [9]	Full catastrophe living	-	2326	41,070
3	Baer (2006) [34]	Using self-report assessment methods to explore facets of mindfulness	Assessment	2298	42,161
4	Bishop (2004) [35]	Mindfulness: a proposed operational definition	Clinical Psychology-Science and Practice	1956	38,294
5	Kabat-Zinn (2003) [2]	Mindfulness-based interventions in context: past, present, and future	Clinical Psychology-Science and Practice	1695	27948
6 *	Segal (2002) [11]	Mindfulness-based cognitive therapy for depression: A new approach to preventing relapse	-	1681	31,151
7	Baer (2003) [37]	Mindfulness training as a clinical intervention: A conceptual and empirical review	Clinical Psychology-Science and Practice	1390	27,313
8	Grossman (2004) [38]	Mindfulness-based stress reduction and health benefits: a meta-analysis	Journal of Psychosomatic Research	1355	25,201
9	Kabat-Zinn (1982) [8]	An outpatient program in behavioral medicine for chronic pain patients based on the practice of mindfulness meditation: Theoretical considerations and preliminary results	General Hospital Psychiatry	1240	23,396
10	Hofmann (2010) [39]	The effect of mindfulness-based therapy on anxiety and depression: A meta-analytic review	Journal of Consulting and Clinical Psychology	1214	24,499
11 *	Kabat-Zinn (1994) [10]	Wherever you go, there you are: Mindfulness meditation in everyday life	-	1154	20,077
12	Shapiro (2006) [42]	Mechanisms of mindfulness	Journal of Clinical Psychology	988	22,373
13	Brown (2007) [41]	Mindfulness: theoretical foundations and evidence for its salutary effects	Psychological Inquiry	975	19,123
14	Baer (2008) [64]	Construct validity of the five-facet mindfulness questionnaire in meditating and nonmeditating samples	Assessment	969	18,801
15 *	Hayes (1999) [63]	Acceptance and commitment therapy: An experiential approach to behavior change	-	946	16,594
16	Teasdale (2000) [12]	Prevention of relapse/recurrence in major depression by mindfulness-based cognitive therapy	Journal of Consulting and Clinical Psychology	933	18,202
17	Holzel (2011) [67]	How does mindfulness meditation work? Proposing mechanisms of action from a conceptual and neural perspective	Perspective on Psychological Science	862	19,634
18	Cohen (1983) [66]	A global measure of perceived stress	Journal of Health and Social Behavior	852	11,765
19	Baer (2004) [65]	Assessment of mindfulness by self-report: The Kentucky inventory of mindfulness skills	Assessment	785	16,396
20	Lutz (2008) [13]	Attention regulation and monitoring in meditation	Trends in Cognitive Science	780	17,447

* Book.

**Table 8:** Top 30 references with the strongest citation bursts.

References	Strength	Begin	End	2000–2021
Brown (2003) [33]	198.32	2005	2011	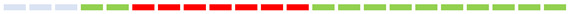
Baer (2006) [34]	191.14	2009	2014	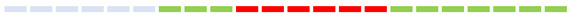
Baer (2003) [37]	156.41	2003	2011	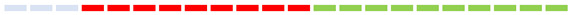
Segal (2002) [11]	152.23	2003	2010	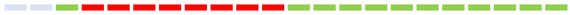
Grossman (2004) [38]	151.87	2006	2012	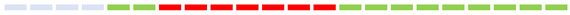
Hofmann (2010) [39]	148.33	2013	2018	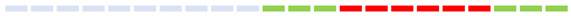
Bishop (2004) [35]	124.1	2006	2012	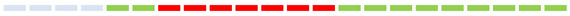
Tang (2015) [68]	116.86	2017	2021	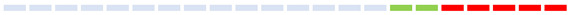
Brown (2007) [41]	113.32	2009	2015	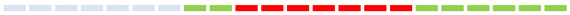
Davidson (2003) [43]	113.22	2004	2011	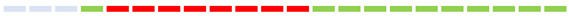
van Dam (2018) [28]	104.59	2019	2021	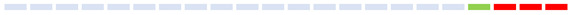
Holzel (2011) [67]	102.54	2015	2019	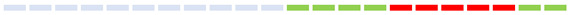
Baer (2004) [65]	95.357	2006	2012	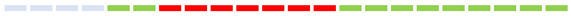
Shapiro (2006) [42]	95.147	2009	2014	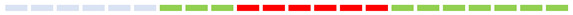
Gu (2015) [69]	93.673	2017	2021	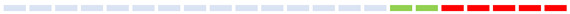
Teasdale (2000) [12]	89.247	2002	2008	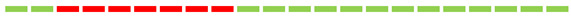
Lutz (2008) [13]	88.544	2010	2016	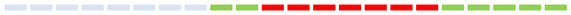
Kabat-zinn (2003) [2]	88.026	2004	2011	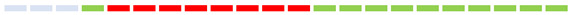
Goyal (2014) [70]	84.49	2016	2021	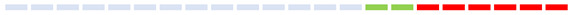
Keng (2011) [71]	81.431	2014	2019	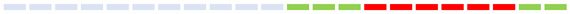
Ma (2004) [72]	81.234	2005	2011	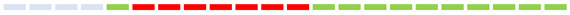
Khoury (2015) [73]	80.751	2017	2021	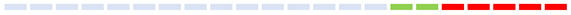
Carmody (2008) [74]	80.172	2010	2015	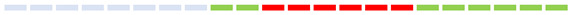
Khoury (2013) [75]	78.538	2016	2021	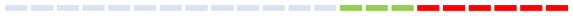
Lazar (2005) [76]	77.052	2007	2013	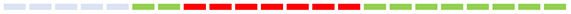
Hayes (2006) [36]	74.695	2007	2013	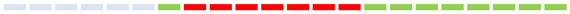
Hayes (2013) [77]	73.719	2016	2021	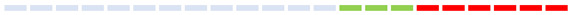
Jha (2007) [78]	73.3	2009	2015	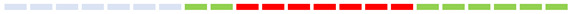
Creswell (2017) [79]	72.008	2018	2021	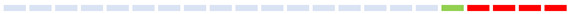
Cahn (2006) [80]	71.744	2008	2013	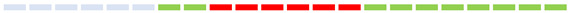

Green color indicates the years since the article was published. Red color indicates the years with citation bursts.

## Data Availability

The datasets generated and/or analyzed during the current study are available from the corresponding author upon reasonable request.

## Appendix A

**Table A1:** Items before and after unification.

NO.	Items	Replace by
1	acceptance and commitment therapy (act)	acceptance and commitment therapy
2	act	acceptance and commitment therapy
3	adolescent	adolescents
4	adult	adults
5	african americans	african-americans
6	african-american	african-americans
7	alcohol dependence	alcohol
8	alcohol-use	alcohol
9	alzheimers-disease	alzheimer’s disease
10	anterior cingulate	anterior cingulate cortex
11	anxiety disorder	anxiety disorders
12	associations	association
13	autonomic nervous system	autonomic nervous-system
14	behavior therapy	behavioral therapy
15	behavior-therapy	behavioral therapy
16	blood pressure	blood-pressure
17	behaviors	behavior
18	body image	body-image
19	borderline personality disorder	borderline personality-disorder
20	brain activity	brain
21	breast cancer	breast-cancer
22	breast-cancer patients	breast-cancer
23	breast-cancer survivors	breast-cancer
24	buddhist	buddhism
25	cancer outpatients	cancer
26	cancer-patients	cancer
27	cardiovascular disease	cardiovascular-disease
28	caregiver	caregivers
29	child	children
30	childbirth	children
31	childhood	children
32	clinical-trials	clinical-trial
33	cognitive behavior therapy	cognitive-behavioral therapy
34	cognitive behaviour therapy	cognitive-behavioral therapy
35	cognitive behavioural therapy	cognitive-behavioral therapy
36	cognitive-behavior therapy	cognitive-behavioral therapy
37	cognitive behavioral therapy	cognitive-behavioral therapy
38	college	college-students
39	college students	college-students
40	commitment	commitment therapy
41	compassion meditation	compassion
42	complementary medicine	complementary and alternative medicine
43	complementary therapies	complementary and alternative medicine
44	construct	construct-validity
45	coping strategies	coping
46	default mode	default mode network
47	default network	default mode network
48	deficit/hyperactivity disorder	deficit hyperactivity disorder
49	depression scale	depression
50	ddepressive symptoms	depression
51	epressive relapse	depression
52	dialectical behavior therapy	dialectical behavior-therapy
53	disabilities	disability
54	disorder	disorders
55	eating behaviors	eating behavior
56	eating-disorders	eating disorders
57	emotion-regulation	emotion regulation
58	emotional regulation	emotion regulation
59	emotions	emotion
60	executive functions	executive function
61	family	families
62	group intervention	group therapy
63	heart rate	heart-rate-variability
64	heart rate variability	heart-rate-variability
65	heart-rate	heart-rate-variability
66	individual differences	individual-differences
67	intellectual disabilities	intellectual disability
68	interventions	intervention
69	loving-kindness	loving-kindness meditation
70	major depressive disorder	major depression
71	mechanism	mechanisms
72	mediator	mediators
73	medical students	medical-students
74	mental health	mental-health
75	metaanalysis	meta-analysis
76	mbsr	mindfulness based stress reduction
77	mindfulness-based stress reduction (mbsr)	mindfulness based stress reduction
78	models	model
79	moderators	moderator
80	multiple-sclerosis	multiple sclerosis
81	music therapy	music-therapy
82	needs	need
83	network	networks
84	older adults	older-adults
85	parent	parents
86	perceptions	perception
87	personality-traits	personality
88	positive emotion	positive emotions
89	posttraumatic stress disorder	posttraumatic-stress-disorder
90	posttraumatic stress	posttraumatic-stress
91	predictor	predictors
92	prediction	predictors
93	primary care	primary-care
94	prostate-cancer outpatients	prostate-cancer
95	psychological intervention	psychological interventions
96	psychological treatment	psychological treatments
97	quality of life	quality-of-life
98	randomised controlled trial	randomized controlled-trial
99	randomized controlled trial	randomized controlled-trial
100	randomized controlled trials	randomized controlled-trial
101	randomized controlled-trials	randomized controlled-trial
102	randomized-controlled-trial	randomized controlled-trial
103	religiosity	religion
104	resting-state	resting state
105	scales	scale
106	schools	school
107	smoking cessation	smoking-cessation
108	strategy	strategies
109	stress reduction intervention	stress reduction
110	stress reduction program	stress reduction
111	stress reduction mbsr	stress reduction
112	student	students
113	style	styles
114	tai-chi	tai chi
115	therapies	therapy
116	thoughts	thought
117	transcendental meditation	transcendental-meditation
118	university students	university-students
119	validity	validation
120	weight loss	weight-loss
121	wellbeing	well-being
122	working memory	working-memory
123	young adults	young-adults
124	zen	zen meditation

## Appendix B

**Table A2:** The 10 most prolific countries/regions (1900–1999).

Rank	Country	Publications	Citations	Citations/Publication
1	USA	328	17,390	53.02
2	England	37	765	20.68
3	Canada	32	699	21.84
4	Germany	20	1935	96.75
5	Australia	17	836	49.18
6	India	13	218	16.77
7	New Zealand	8	53	6.63
8	France	7	5	0.71
9	Spain	6	111	18.5
10	Switzerland	6	198	33

## Appendix C

**Table A3:** The top 10 cited articles (1900–1999).

Rank	First Author	Year	Journal	Article	Citations
1	Kabat-zinn, J	1982	General Hospital Psychiatry	An outpatient program in behavioral medicine for chronic pain patients based on the practice of mindfulness meditation—theoretical considerations and preliminary-results	1804
2	Kirschbaum, C	1994	Psychoneuroendocrinology	Salivary cortisol in psychoneuroendocrine research—recent developments and applications	1427
3	Weick, KE	1999	Research in Organizational Behavior	Organizing for high reliability: Processes of collective mindfulness	995
4	Kabat-zinn, J	1992	American Journal of Psychiatry	Effectiveness of a meditation-based stress reduction program in the treatment of anxiety disorders	939
5	Epstein, RM	1999	JAMA-Journal of the American Medical Association	Mindful practice	766
6	Shapiro, SL	1998	Journal of Behavioral Medicine	Effects of mindfulness-based stress reduction on medical and premedical students	762
7	Miller, JJ	1995	General Hospital Psychiatry	3-year follow-up and clinical implications of a mindfulness mediation-based stress reduction intervention in the treatment of anxiety disorders	491
8	Wallace, RK	1970	Science	Physiological effects of transcendental meditation	391
9	Kabat-zinn, J	1998	Psychosomatic Medicine	Influence of a mindfulness meditation-based stress reduction intervention on rates of skin clearing in patients with moderate to severe psoriasis undergoing phototherapy (UVB) and photochemotherapy (PUVA)	374
10	Astin, JA	1997	Psychotherapy and Psychosomatics	Stress reduction through mindfulness meditation—Effects on psychological symptomatology, sense of control, and spiritual experiences	329
